# Validating Foundation Models for Automated Cattle Detection

**DOI:** 10.3390/s26165074

**Published:** 2026-08-10

**Authors:** Petra Pejić, Andrej Bošnjak, Robert Cupec, Emmanuel Karlo Nyarko, Josip Job, Boris Lukić

**Affiliations:** 1 Faculty of Electrical Engineering, Computer Science and Information Technology Osijek, 31000 Osijek, Croatia; andrej.bosnjak@ferit.hr (A.B.); robert.cupec@ferit.hr (R.C.); karlo.nyarko@ferit.hr (E.K.N.); josip.job@ferit.hr (J.J.); 2 Faculty of Agrobiotechnical Sciences Osijek, 31000 Osijek, Croatia; boris.lukic@fazos.hr

**Keywords:** cattle detection, automated annotation, foundation models, SAM, YOLO

## Abstract

Automated monitoring of cattle behavior through computer vision requires robust detection as a foundational step for tracking, re-identification, and behavior analysis. However, training accurate detection models typically demands extensive manually annotated datasets, creating a significant bottleneck for scaling cattle monitoring systems. In this work, we investigate whether automated annotation using foundation models can reliably replace manual labeling for cattle detection tasks. We introduce EMA (Extensive Mitrovac Annotations), a dataset of barn images with manually annotated cows with oriented bounding boxes including head orientation, posture labels (standing/lying), and visibility status (whole/partially visible). We systematically compare manualy annotated oriented bounding boxes with those generated by the Segment Anything Model 3 (SAM 3), demonstrating high agreement between automated and ground truth annotations. Furthermore, we train YOLO11-OBB detectors on both manual and SAM-generated annotations, showing that models trained on automated annotations achieve comparable performance to those trained on manual labels when evaluated on our ground truth test set. Our analysis reveals that only a small fraction of SAM-annotated data is sufficient to achieve high detection accuracy, proving the feasibility of automated annotation at scale. These findings suggest that foundation models show promise for generating training data in cattle detection pipelines under controlled conditions, potentially reducing annotation costs and supporting scalable deployment of monitoring systems. The EMA dataset and trained models are publicly available to support further research in precision livestock farming.

## 1. Introduction

Monitoring cattle behavior on farms provides essential information about animal health and welfare, offering early indicators of lameness, inflammatory conditions, calving complications, and feeding abnormalities. In large-scale farming operations, continuous human observation is impractical and often leads to delayed problem detection. To enhance efficiency, animal welfare, and productivity, modern farms are adopting computer vision-based monitoring systems that can detect, track, identify, and analyze cattle behavior non-invasively, without interfering with natural animal activities.

Camera-based monitoring offers significant advantages over conventional systems such as wearable sensors or RFID tags, which require substantial labor for installation and maintenance, are prone to loss or damage, and may cause discomfort to animals [1]. In contrast, fixed cameras enable continuous observation without disturbing cattle, as animals typically disregard their presence [2].

A comprehensive cattle monitoring pipeline consists of several sequential stages: detection (locating cattle and their behavioral states within frames), tracking (maintaining individual identities across frames), re-identification (recognizing individuals across different camera views), and behavior analysis (monitoring activities such as walking, standing, lying, feeding, or drinking) [3]. Detection serves as the critical first stage upon which all subsequent tasks depend. However, training robust detection models typically requires extensive manually annotated datasets—a time-consuming and expensive process that creates a significant bottleneck for deploying cattle monitoring systems at scale.

Modern object detection approaches, such as YOLO (You Only Look Once) [4], have demonstrated outstanding performance across various visual domains and can be fine-tuned on farm-specific datasets to adapt to the unique characteristics of barn environments. These detectors output bounding boxes (either axis-aligned or oriented), class labels, and confidence scores, providing an optimal balance of accuracy, efficiency, and real-time deployability. Oriented bounding boxes (OBB) are particularly suitable for cattle detection, as they better conform to animal pose, reduce background content, and can encode additional information such as head orientation—critical for behavior analysis. Despite these advances, the reliance on manual annotation remains a primary obstacle to scaling cattle monitoring systems.

Recent foundation models, such as SAM 3 (Segment Anything Model) [5], offer the potential to automate or semi-automate the annotation process. However, a critical question remains: Can automatically generated annotations from models like SAM 3 serve as reliable training data for task-specific detectors, effectively replacing expensive manual annotation? Answering this question is essential for establishing scalable, cost-effective cattle monitoring pipelines.

In this paper, we address this question through a systematic evaluation of automated annotation for cow detection. We introduce EMA (Extensive Mitrovac Annotations), a large-scale dataset of images with manually annotated oriented bounding boxes, captured during daily activities in a barn environment at Mitrovac farm. We then compare manual annotations with those generated by SAM 3, demonstrating that SAM 3 produces high-quality annotations suitable for training detection models. Furthermore, we train YOLO11-OBB detectors on both manual (EMA) and automated (SAM 3) annotations, showing that models trained on SAM 3-generated data achieve comparable performance to those trained on manual annotations when evaluated on the manually annotated EMA test set. Finally, we demonstrate that even a small fraction of the SAM 3-annotated dataset is sufficient to train highly accurate detectors, proving the feasibility of large-scale automated annotation.

Our findings suggest that unsupervised or automated annotation methods show potential as a foundation for cattle detection systems under the tested conditions, offering opportunities to reduce the annotation burden. However, they do not yet establish general reliability across different farms, breeds, seasonal variations, camera placements, nighttime environments, lens contamination, diverse barn structures, or more extreme occlusion scenarios. Further research is needed to validate their reliability across diverse farming environments. This work offers practical guidance for researchers and practitioners seeking to develop cost-effective, large-scale cattle monitoring solutions.

The main contributions of this work are:

- Validation of automated annotation: A comprehensive evaluation demonstrating that SAM 3-generated annotations achieve high agreement with manual ground truth, establishing their reliability for cattle detection tasks.
- EMA dataset: A large-scale, manually annotated dataset consisting of 6295 cow images with 25,014 oriented bounding boxes of fully visible cows, and a subset of 1096 images with 13,119 oriented bounding boxes covering both fully and partially visible cows. Manual annotations additionally include head orientations, posture labels, and visibility status—attributes not typically provided by automated tools. These supplementary features were not evaluated against automatic annotations, as the SAM 3 model outputs only object masks (which we converted to oriented bounding boxes) without semantic attributes.
- Large-scale automatically annotated dataset: 6295 cow images annotated using SAM 3, demonstrating the scalability of automated annotation methods for real-world barn environments.
- Training data requirements analysis: A systematic study showing that YOLO11-OBB models trained on SAM 3 annotations achieve comparable performance to those trained on manual annotations, and that only a small fraction of automated annotations is needed to reach high detection accuracy.

The EMA dataset, along with our comparative analysis of manual and automated annotations, serves as a valuable resource for the computer vision and precision agriculture communities, providing benchmarks for validating novel detection algorithms and facilitating transfer learning for cattle monitoring applications.

The paper is structured as follows. Section 1 motivates the need for automated annotation in cattle monitoring and establishes that foundation models can be trusted as annotation tools, followed by Section 2 which analyzes related research in the scope of cattle datasets and methods for their annotation and detection. Section 3 introduces the EMA dataset, describing image acquisition, manual annotation methodology, and dataset organization. Section 4 presents the methods, detailing SAM 3 for automated annotation and YOLO11-OBB for detection training. Section 5 provides comprehensive experimental evaluation, including SAM 3 parameter selection, matching criteria development, comparison of YOLO models trained on manual vs. automated annotations, and analysis of training data size requirements. Section 6 interprets the findings, discusses practical implications, and outlines limitations and future work. Finally, Section 7 summarizes the key contributions and emphasizes the impact on precision livestock farming.

## 2. Related Research

### 2.1. Datasets

Public datasets are essential for developing computer vision systems for farm monitoring because they enable objective comparison and benchmarking across approaches, yet most published datasets are task oriented and reflect specific capture setups. As a result, datasets differ substantially in the conditions under which data are collected and in what they can support experimentally, including camera viewpoint (top down dorsal, angled, side, close up, or aerial), recording environment and farm layout (for example, coverage of milking areas, corridors, barn aisles, and differences in infrastructure such as automatic milking systems, rotary stations, or herringbone parlors), and illumination (day versus night, indoor versus outdoor, and artificial lighting). They also vary in breed composition, optical properties such as lens distortion, and the type and strength of annotations, which directly affect suitability for detection, tracking, identification, or re-identification and how well results transfer to a target deployment. Dataset scale is another frequent constraint: collections with only a small number of individuals can support controlled experiments but may not reflect commercial herd sizes, so despite the apparent variety in the literature, relatively few public datasets match a given application scenario or cover realistic conditions across tasks. To make these differences concrete, several representative public cow datasets and recent releases are summarized below, reporting capture viewpoint, environment, dataset scale, and the type of human verified annotations provided.

Several publicly available datasets address cattle detection, localization, and identification from overhead or top-down viewpoints. OpenCows2020 [6] combines indoor barn cameras and outdoor UAV footage with axis-aligned bounding boxes (AABB), providing 7043 images for detection and an identification subset for 46 cows. Cows2021 [7] offers 10,402 barn images with oriented bounding boxes (OBB) capturing head-to-tail direction for 186 cows, though boxes exclude head, neck, legs, and tail. MultiCamCows2024 [8] provides 101,329 images from multi-camera barn surveillance focused on re-identification using tracklets but lacks manually verified per-frame detection labels. CVB [9] contains 502 outdoor field clips with AABB and behavior labels for 8 cows across four cameras. CBVD-5 [10] provides 687 barn surveillance segments with AABB and five behavior classes for 107 cows. The dataset in [11] offers  1.7 million frames from milking station footage with frame-level activity labels rather than bounding boxes. As highlighted in [12], most annotated cattle datasets remain inaccessible, creating barriers to research progress. Our EMA dataset addresses this gap by providing publicly available, manually verified oriented bounding boxes for fully and partially visible cattle, complementing existing resources with detection-focused annotations suitable for comprehensive barn monitoring. The recently released COLO dataset [13] provides 1254 images with 11,818 cow instances for free-stall barn detection, emphasizing the importance of view angle diversity. Our EMA dataset complements this contribution by providing oriented bounding boxes and additional behavioral annotations across a larger image collection. The summary and comparison of the above mentioned datasets is given in Table 1.

### 2.2. Automatic Annotation with Foundation Models and Detectors

Extensive human annotation remains the most reliable way to obtain ground truth for training and evaluating neural network models, but it is labor intensive, costly, and repetitive. To scale dataset creation, several annotation strategies have been adopted, including active learning to prioritize the most informative samples for labeling, model assisted annotation followed by human correction, semi supervised learning with pseudo labels, and weak supervision based on heuristic labeling functions. Moreover, recent models trained on broad and diverse data, often referred to as foundation models, further reduce manual effort by enabling prompt guided annotation. In particular, SAM 3 [5] produces object masks from simple prompts, which can serve as a strong starting point for segmentation or for deriving bounding boxes that can then be checked or corrected. Authors in [14] describe a semi automated pipeline for cattle video annotation that combines YOLOv8 detection with ByteTrack tracking to generate coordinates and identity codes, followed by manual labeling of cropped clips with behavior categories. Authors in [15] propose an automated workflow for plant leaf segmentation, where SAM generated leaf masks are converted into YOLOv8 compatible bounding boxes to reduce or eliminate manual box labeling during dataset construction. A similar detector plus SAM style approach is also reported for dairy cow video segmentation in SideCow VSS [16], where YOLOv11 proposals and SAM 2 masks are refined through expert verification to produce final pixel annotations.

Recent work has advanced cattle detection by incorporating attention mechanisms into YOLO architectures to handle complex real-world conditions [17], achieving improved generalization across diverse indoor and outdoor environments. Our work complements these architectural improvements by addressing the annotation bottleneck through foundation model-based automated labeling. Beyond detection and tracking, AI-based cattle monitoring extends to specialized welfare applications such as automated pain assessment through facial expression analysis [18], demonstrating the broader potential of computer vision in precision livestock farming.

### 2.3. Evaluation of Automatic Annotation

To evaluate the quality of SAM 3-generated annotations, we employ standard object detection metrics based on bounding box overlap. The most common metrics are precision, recall, F1 score, and mean Average Precision (mAP), computed by matching predicted and ground truth boxes at specified IoU (Intersection over Union) thresholds [15].

However, IoU alone can be problematic when comparing SAM 3 segmentation-based boxes with manual annotations due to systematic box tightness differences. To address this, we complement IoU with asymmetric overlap measures: ${\mathrm{IoA}}_{G}=\left|S\cap G|/|G\right|$ (coverage of ground truth by SAM 3) and ${\mathrm{IoA}}_{S}=\left|S\cap G|/|S\right|$ (tightness of SAM 3 relative to ground truth), where *S* is the SAM 3 prediction and *G* is the ground truth box [19]. We also employ Intersection over Minimum area IoM=|S∩G|/min(|S|,|G|), a containment-oriented measure less sensitive to small localization shifts than IoU alone [20,21].

This multi-faceted evaluation approach enables comprehensive assessment of SAM 3 annotation reliability across different aspects of box quality.

## 3. EMA Dataset

The EMA (Extensive Mitrovac Annotations) dataset was collected to support the development and evaluation of automated cattle detection systems in realistic barn environments. This section describes the image acquisition process, the manual annotation methodology, and the dataset structure.

### 3.1. Image Acquisition

Images in 4K (3840 × 2160) resolution were captured at Belje Mitrovac farm over a two-week period in 2025, using a multi-camera surveillance system installed throughout the barn facility. Although 22 cameras were deployed overall, we selected 12 cameras positioned in areas where cattle most frequently congregate, ensuring high-quality observational data. The multi-camera setup provides diverse viewpoints, varying cow orientations, and a wide range of lighting conditions, enhancing the dataset’s representativeness for real-world deployment scenarios.

To ensure adequate visibility and annotation quality, images were sampled from periods with sufficient illumination, typically between 5:00 a.m. and 8:00 p.m. From the continuous video streams, we extracted frames at regular intervals (approximately every 500th frame). Of the initially 8000 extracted images, 6295 were suitable for annotation and constitute the final dataset, while the remaining images were manually flagged as unusable due to poor visibility (insufficient lighting, lens distortion, occlusion, or other quality issues) and excluded from further processing. This sampling strategy balances temporal diversity while avoiding excessive redundancy between consecutive frames.

All images underwent lens distortion correction prior to annotation to ensure geometric accuracy. All subsequent annotations—both manual and automatic—were performed on these corrected images.

### 3.2. Manual Annotation Process

To create high-quality ground truth annotations, we developed a custom annotation tool optimized for efficient oriented bounding box labeling. The annotation workflow proceeds as follows:

1. The annotator is presented with an image and clicks multiple points (typically 4–8) around each cow’s perimeter.
2. The tool automatically computes the minimum-area oriented bounding box enclosing these points and displays it for verification.
3. The annotator selects which side of the bounding box corresponds to the cow’s head orientation.
4. The annotator assigns a posture label: *standing* or *lying*.
5. For partially visible cows (e.g., at image boundaries or behind obstacles), the annotator sets a visibility flag.
6. This process repeats for all cows in the image.
7. If an image contains no cows or is corrupted, the annotator marks it accordingly.

**Figure 1:**
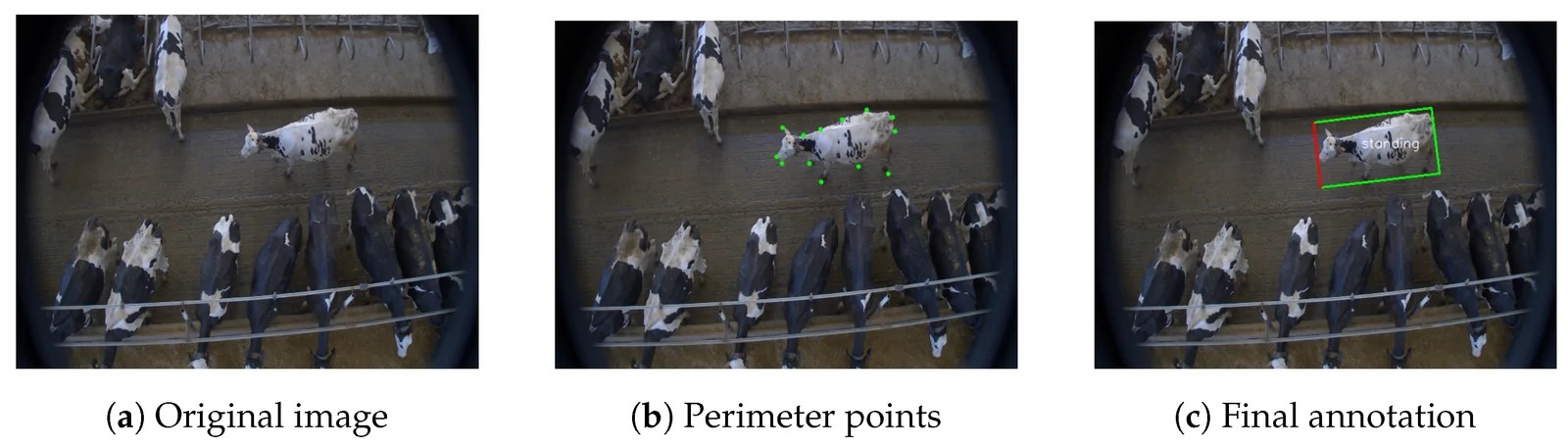
Manual annotation workflow: (**a**) original barn image, (**b**) annotator-selected points defining cow perimeter, (**c**) computed oriented bounding box with head orientation (indicated side) and posture label.

Figure 1 illustrates this process. The point-based interface enables rapid annotation while ensuring tight-fitting oriented bounding boxes that minimize background inclusion.

Each annotation is stored in JSON format with the following metadata:

- Image filename.
- Oriented bounding box: four vertex coordinates and center point.
- Head side: coordinates of the two vertices adjacent to the cow’s head.
- Orientation angle (in degrees).
- Posture label: *standing* or *lying*.
- Visibility flag: *whole* (fully visible) or *partial* (partially visible).
- Validity flag: indicates images without cows or with data corruption.

This comprehensive annotation format enables full reconstruction and visualization of bounding boxes, allowing annotators to verify their work and facilitating quality control through visual inspection. Note that the experiments performed in this paper validate only the cattle detection performance of automated annotation methods. The additional annotations (head orientation, posture, visibility) represent supplementary dataset contributions that enable future work beyond the scope of this study.

### 3.3. Dataset Structure and Composition

The EMA dataset consists of images with multiple annotation variants to support different research needs and comparative analysis. The dataset is organized into the following components:

#### Manual Annotations (Ground Truth)

- EMA_6295: Contains manual annotations for 6295 images, focusing exclusively on fully visible cows, resulting in 25,014 oriented bounding boxes. These annotations were initially created to train a YOLO detector before we recognized that SAM 3 automatically annotates both fully and partially visible cows. To preserve this substantial annotation effort (approximately 220 h of expert labor) and provide valuable training data to the research community, we include this subset in the public release. However, for direct comparison with SAM 3 annotations in our experimental evaluation, we use only the EMA_1096 subset described below, which includes both fully and partially visible cows and is therefore directly comparable to SAM 3 output. Additionally, the annotation time invested in EMA_6295 provides important benchmark data for comparing manual versus automated annotation costs—a key contribution of this work.
- EMA_1096: A carefully annotated subset of 1096 images that includes both fully and partially visible cows, yielding 13,119 total annotations. Annotations in this subset, often referred to as ground truth in this paper, captures more challenging scenarios including edge cases, occlusions, and boundary conditions, and serves as the primary evaluation set for all experiments reported in this paper.

#### Automatic Annotations

To evaluate the reliability of automated annotation methods, we generated automatic annotations using SAM 3 (methodology detailed in Section 4). These are provided in additional sets:

- SAM_6295.
- SAM_1096.

The 1096-image subsets (EMA_1096 and SAM_1096) share identical source images, enabling direct quantitative comparison between manual and automatic annotations. This comparison is central to our evaluation of whether automated methods can serve as reliable alternatives to manual annotation. The difference between EMA_6295 and SAM_6295 is that EMA_6295 contains only fully visible cows’ annotations, while the latter contains annotations of both fully and partially visible cows, thus they cannot be directly compared. Note that SAM_1096 is a subset of SAM_6295, comprising the same 1096 images as EMA_1096. We provide SAM_1096 separately to enable direct comparison between manual and automated annotations on identical images.

#### Fine-Tuned Models

The dataset release includes YOLO model checkpoints trained on both manual ground truth annotations (EMA_1096) and automatic annotations (SAM_1096 and SAM_6295) provided by SAM 3, enabling reproducibility and facilitating transfer learning for related cattle monitoring tasks.

Note that EMA_1096 and SAM_1096 share identical source images, divided into train/val/test splits using the same partitioning. This enables direct comparison: models trained on the training subset with either EMA_1096 or SAM_1096 annotations are both evaluated on the same test subset using ground truth EMA_1096 annotations, ensuring a controlled comparison between manual and automated annotation sources.

### 3.4. Dataset Statistics

Table 2 summarizes key statistics of the EMA dataset components.

The dataset exhibits natural variability in cow density, posture distribution, and environmental conditions typical of barn environments.

Manual annotation process requires approximately 25 h for annotating 400 images, which in total would take around 400 h for annotating 6295 images with both fully and partially visible cows. Automated methods using SAM 3 can process the same volume in approximately 1 h with comparable quality and crucially, zero human effort. This elimination of manual annotation labor enables scalable dataset creation that would otherwise be prohibitively expensive.

Inter-annotator agreement was evaluated on 404 images containing approximately 5100 oriented bounding boxes. Two annotations were considered a match if their Intersection over Union (IoU) was at least 0.5 or their Intersection over Minimum (IoM) was at least 0.9. Visual inspection showed that the IoM criterion was necessary because the second annotator consistently produced tighter bounding boxes than the first annotator. Consequently, many boxes described the same object and had nearly identical locations and orientations, despite having insufficient overlap to satisfy the IoU criterion alone. The IoM criterion identified an additional 349 valid matches. Overall, the annotations achieved a symmetric F1 score of 0.944, with a mean IoU of 0.705. Although only 42.4% of the matched boxes reached an IoU of at least 0.75, the mean IoM was 0.961, and the mean difference in bounding-box orientation was only 3.94°. These results indicate strong agreement between the annotators regarding object location and orientation, while the remaining differences primarily reflect variation in how tightly the object boundaries were annotated.

### 3.5. Data Availability

The complete EMA dataset, including all images, manual and automatic annotations and fine-tuned model checkpoints is publicly available at: https://puh.srce.hr/s/gC2RMdDb2rga2FC (accessed on 21 July 2026).

We encourage the research community to use this dataset for developing and benchmarking cattle detection and behavior analysis systems.

## 4. Methodology

This section describes the methods and models used in our study: SAM 3 for automated annotation and YOLO11-OBB for detection training.

### 4.1. Segment Anything Model 3 (SAM 3)

The Segment Anything project introduced promptable image segmentation: given an input image and a prompt specifying an object or region of interest, the model predicts a segmentation mask corresponding to that prompt [22]. Prompts may be sparse (e.g., points, bounding boxes) or dense (input masks), supporting both interactive annotation and automatic pipelines where prompts are provided by other components such as detectors.

Architecturally, SAM decomposes computation into three components: an image encoder, a prompt encoder, and a lightweight mask decoder. The image embedding is computed once per image, and subsequent prompts are encoded and decoded efficiently, enabling rapid iteration over multiple prompts. To handle prompt ambiguity, SAM can output multiple candidate masks, each with a predicted quality score to support selection of the most plausible mask. The original SAM model was trained using a model-in-the-loop data engine that produced the SA-1B dataset (over one billion masks on eleven million images), enabling broad generalization without retraining for each downstream application.

While SAM was designed for static images, subsequent versions extended its capabilities. SAM 2 extended the prompt-based interface from images to videos by introducing a streaming memory mechanism for temporal propagation and refinement, trained on large-scale video segmentation data [23]. SAM 3, used in this work, further extends prompting to concept-based prompts (e.g., short noun phrases) and targets retrieving all matching instances while maintaining identities in video through a unified detector and memory-based tracker design [5]. Specifically, this work uses the SAM 3 image model from sam3==0.1.0, based on the official facebookresearch/sam3 implementation, and loaded from the default Hugging Face checkpoint facebook/sam3/sam3.pt.

In our application, we leverage SAM 3’s ability to automatically detect and segment all cow instances in an image without explicit per-instance prompting, making it well-suited for generating training annotations at scale. SAM 3 segmentation masks were generated using the text prompt: *cow*.

### 4.2. YOLO11-OBB for Detection

To quantify how annotation source affects detector training, we employ YOLO11-OBB (You Only Look Once version 11 with Oriented Bounding Box output), a standard object detector specifically designed for oriented bounding box detection. YOLO11-OBB represents each detection as four corner points $\left(x_{1},y_{1},\ldots ,x_{4},y_{4}\right)$ together with a class label and confidence score [4]. In this work, the official Ultralytics YOLO11-OBB model is used, whose released checkpoints are pretrained on the DOTA v1.0 aerial image dataset for oriented object detection [24].

Training, validation, and test image splits follow a 70/15/15 ratio of 1096 images (767/164/165). The only difference between experiments is the training annotation source (EMA_1096 vs. SAM_1096); evaluation is always performed on the same test split using ground truth EMA_1096 annotations.

The complete hardware and software configuration, together with all training parameters, is provided in Table 3. All models were trained for 100 epochs. The default Ultralytics early-stopping patience of 100 epochs was retained. Because the patience was equal to the maximum number of epochs, early stopping was effectively inactive, and all training runs were completed without early stopping triggering.

## 5. Experimental Evaluation

This section describes the evaluation methodology, parameter selection for SAM 3, and results comparing manual vs. automated annotations for training YOLO detection models.

### 5.1. SAM 3 Annotation Quality Assessment

To properly evaluate SAM 3 annotations, we must define what constitutes a valid detection. Because SAM 3 produces pixel-level segmentations, it can return masks corresponding to small visible parts of cows that may be marginal or uninformative for downstream analysis. Such partial fragments contain limited visual information and can inflate detection counts without improving practical utility. Additionally, SAM 3 outputs a confidence score for each predicted instance, computed as the product of an image-level recognition score (whether the prompted object is present in the image) and an instance-level localization score (how strongly the region matches the prompt). SAM 3 annotations were generated automatically by applying SAM 3 to each image using the text prompt *cow*. For every predicted cow instance, SAM 3 outputs a segmentation mask together with a confidence score. Since the rest of our annotation and detection pipeline is based on oriented bounding boxes, each predicted mask was converted into an oriented bounding box by fitting the minimum-area rectangle around the mask pixels. The resulting box was stored as four corner points, together with the corresponding SAM 3 confidence score.

#### 5.1.1. Parameter Selection: Confidence and Minimal Relative Area

To optimize SAM 3 annotation quality, we evaluated three confidence thresholds (0.6, 0.75, 0.9) in combination with a minimal relative area (MRA) constraint. The MRA removes detections whose area falls below a predefined fraction of the largest detected object in the same image, effectively filtering spurious small detections while preserving valid partial cow observations.

MRA thresholds were tested at values 0.05, 0.1, 0.15, and 0.2, representing the minimum allowed area relative to the largest detected object. These values were selected empirically as a simple heuristic that eliminates very small detections while preserving those corresponding to visible cows. This analysis was performed on the EMA_1096 subset and should be interpreted as threshold calibration rather than an independent final test. Table 4 summarizes evaluation results in terms of precision, recall, F1 score, and average precision (AP). Each threshold combination was evaluated using an overlap-based matching criterion (described in Section 5.1.2).

The results reveal a clear tradeoff between precision and recall as MRA increases. For all confidence thresholds, larger MRA values increase precision but decrease recall, indicating that while small detections often correspond to false positives, some valid detections are also removed.

The highest F1 score (0.929) is achieved with confidence 0.75 and MRA 0.05, indicating the best balance between precision and recall. In contrast, the highest AP (0.938) is obtained with confidence 0.6 without the MRA constraint, suggesting the best overall detection ranking performance. When F1 score and average precision are considered equally important, the most balanced setting is confidence 0.6 with MRA 0.05.

A confidence threshold of 0.9 produces extremely high precision (>0.99) but severely degrades recall (0.59), making it impractical for automated annotation where completeness is important. Based on these results, all subsequent SAM 3 annotations use confidence threshold 0.6 and MRA threshold 0.05. Because the same EMA_1096 subset was used for this calibration, the values in Table 4 are not reported as independent test-set performance, but as a sensitivity analysis used to fix the SAM 3 filtering parameters before the downstream experiments.

**Figure 2:**
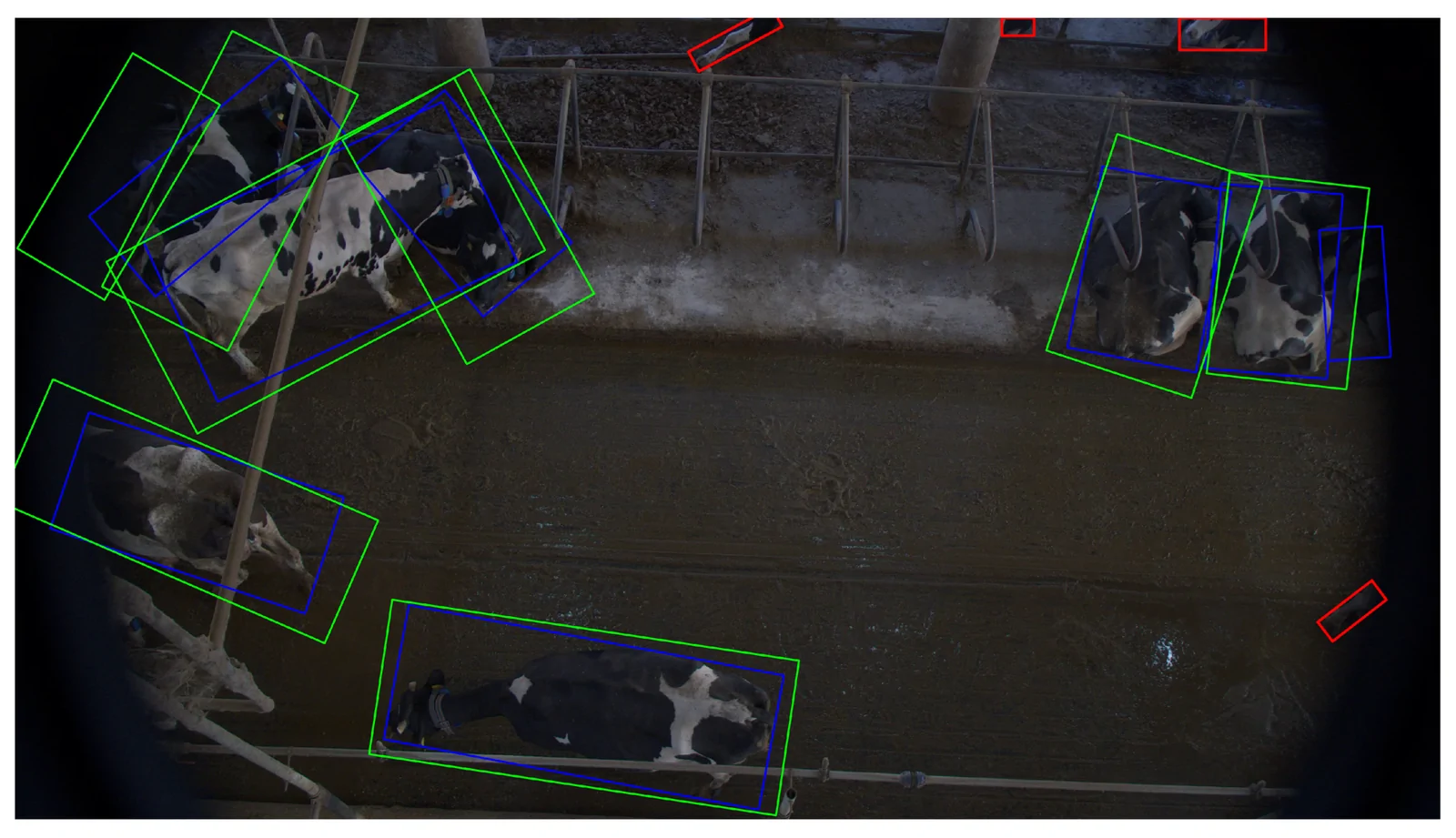
Example demonstrating the effect of the minimal relative area (MRA) constraint. Green bounding boxes represent ground truth annotations. Blue bounding boxes represent SAM annotations that have passed the confidence threshold and MRA constraint, while red bounding boxes do not pass the MRA constraint, showing how small bounding boxes that are not useful for this task are filtered out.

Figure 2 illustrates how the MRA constraint removes small, uninformative detections while preserving valid cow instances.

#### 5.1.2. Matching Criterion: Combining IoU and IoM

A critical methodological decision is defining when a SAM 3 detection matches a ground truth EMA annotation. The standard Intersection over Union (IoU) metric alone can be problematic for comparing SAM 3 and manual annotations due to systematic differences in box tightness: SAM 3 produces pixel-level segmentations that result in tighter bounding boxes, while manual annotations may include more background. When a tight predicted box lies largely inside a larger reference box, the union area is dominated by the reference, potentially pushing IoU below the matching threshold despite substantial overlap.

To address this, we applied an additional containment-based criterion: Intersection over Minimum (IoM), defined as the intersection area divided by the area of the smaller bounding box:

$$\mathrm{IoM}\left(B_{\mathrm{pred}},B_{\mathrm{GT}}\right)=\frac{\mathrm{Area}(B_{\mathrm{pred}}\cap B_{\mathrm{GT}})}{\min(\mathrm{Area}\left(B_{\mathrm{pred}}\right),\mathrm{Area}\left(B_{\mathrm{GT}}\right))}$$

where $B_{\mathrm{pred}}$ is the predicted OBB generated from the SAM 3 mask and $B_{\mathrm{GT}}$ is the ground truth annotation.

A detection is considered a true positive if it satisfies either:

- IoU ≥ 0.5.
- IoM ≥ threshold (to be determined).

**Figure 3:**
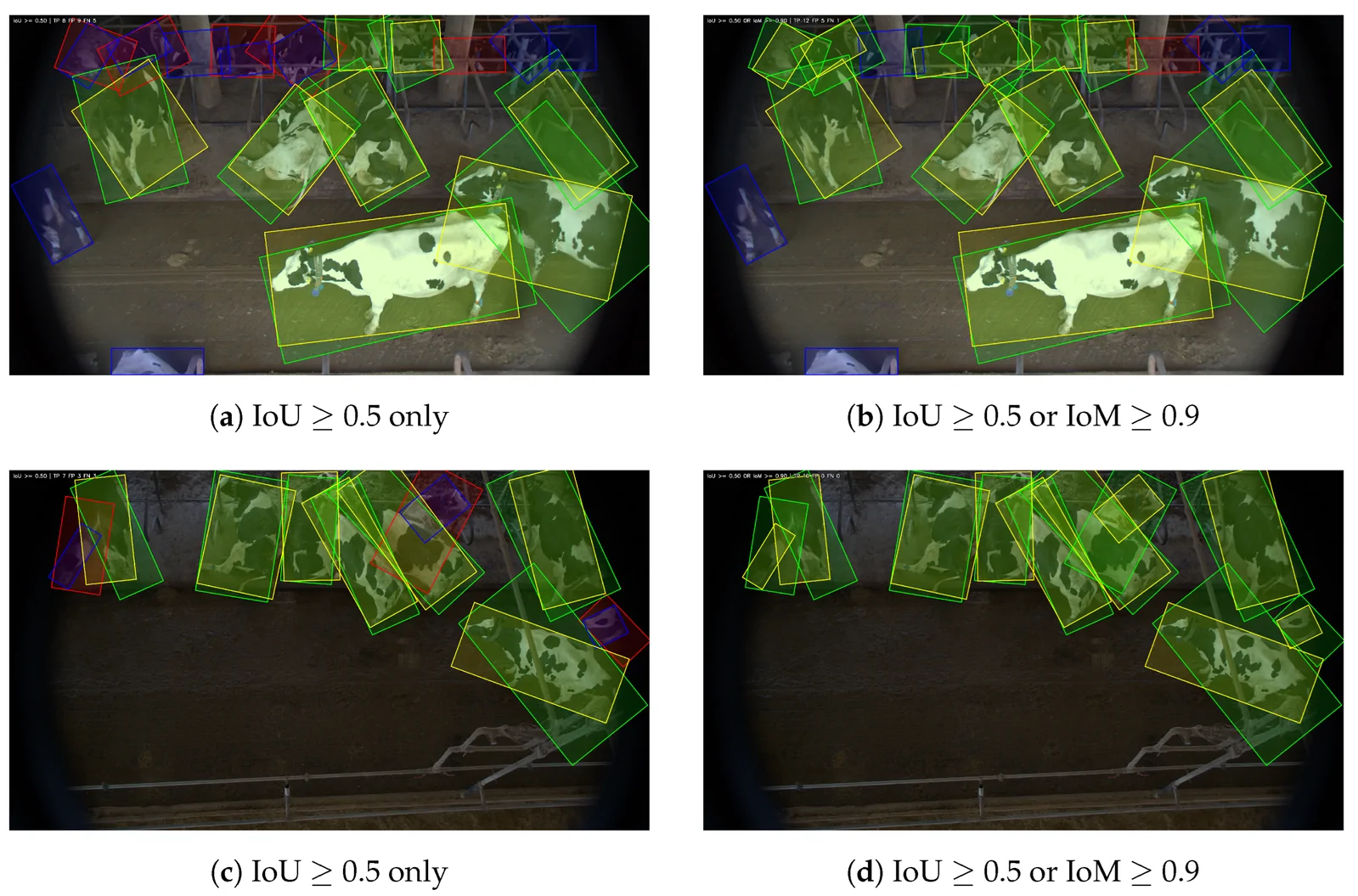
Comparison of matching criteria for two pairs of images. The combined IoU/IoM criterion recovers valid detections that would otherwise be penalized due to bounding box tightness differences. **Legend:** Yellow = correctly matched EMA ground-truth OBB (True Positive). Green = correctly matched SAM prediction OBB (True Positive). Red = EMA ground-truth OBB missed by SAM (False Negative). Blue = SAM false-positive OBB (False Positive).

Figure 3 illustrates the impact of this combined criterion. Using IoU ≥ 0.5 alone leaves 4 in Figure 3a and 3 in Figure 3c detections unmatched (counted as false positives), even though each prediction is almost fully contained within the reference box. Adding the IoM ≥ 0.90 criterion reclassifies all 7 cases as true positives, yielding a matching rule more consistent with the intended notion of detection correctness when systematic box tightness differences exist.

All subsequent evaluations use IoU ≥ 0.5 or IoM ≥ 0.90 as the matching criterion, representing a compromise between recovering plausible matches caused by box tightness differences and avoiding overly permissive weak containment matches.

#### 5.1.3. Edge Cases and Limitations

While the combined IoU/IoM criterion substantially improves matching quality, certain edge cases warrant discussion.

##### Overlapping Cows

**Figure 4:**
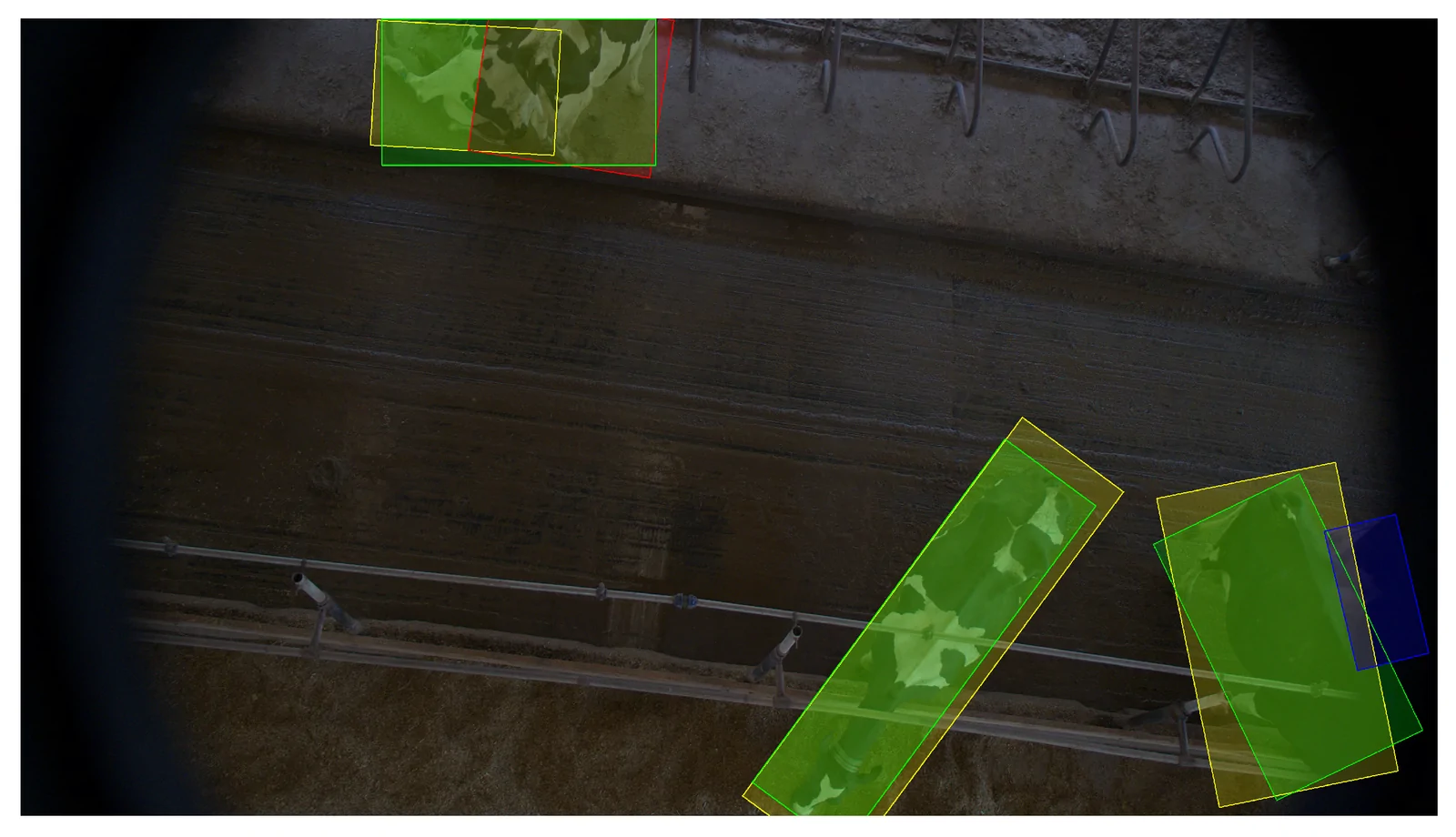
Edge case illustrating a limitation of the IoM criterion. Top: overlapping cows where SAM 3 produces a single detection covering both ground truth boxes, incorrectly counted as one TP instead of one FP and two FNs. **Legend:** Yellow = correctly matched EMA ground-truth OBB (True Positive); Green = correctly matched SAM prediction OBB (True Positive); Red = EMA ground-truth OBB missed by SAM (False Negative); Blue = SAM false-positive OBB (False Positive).

One limitation of incorporating IoM occurs when cows overlap significantly. Typically, SAM 3 successfully segments overlapping cows into separate instances. However, in rare cases, it fails and generates a mask that spans across two cows, resulting in a single bounding box encompassing both animals. Under IoU-only matching, this would result in two false negatives (two ground truth cows not detected) and one false positive (one erroneous prediction). With the IoM criterion, this becomes one false negative and one true positive, as the SAM 3 box contains one of the ground truth boxes sufficiently. Figure 4 illustrates this scenario (in the top of the image). While this represents a theoretical limitation, such cases are extremely rare in practice, and the overall benefits of IoM far outweigh this negligible downside.

##### High Occlusion and Ambiguous Poses

**Figure 5:**
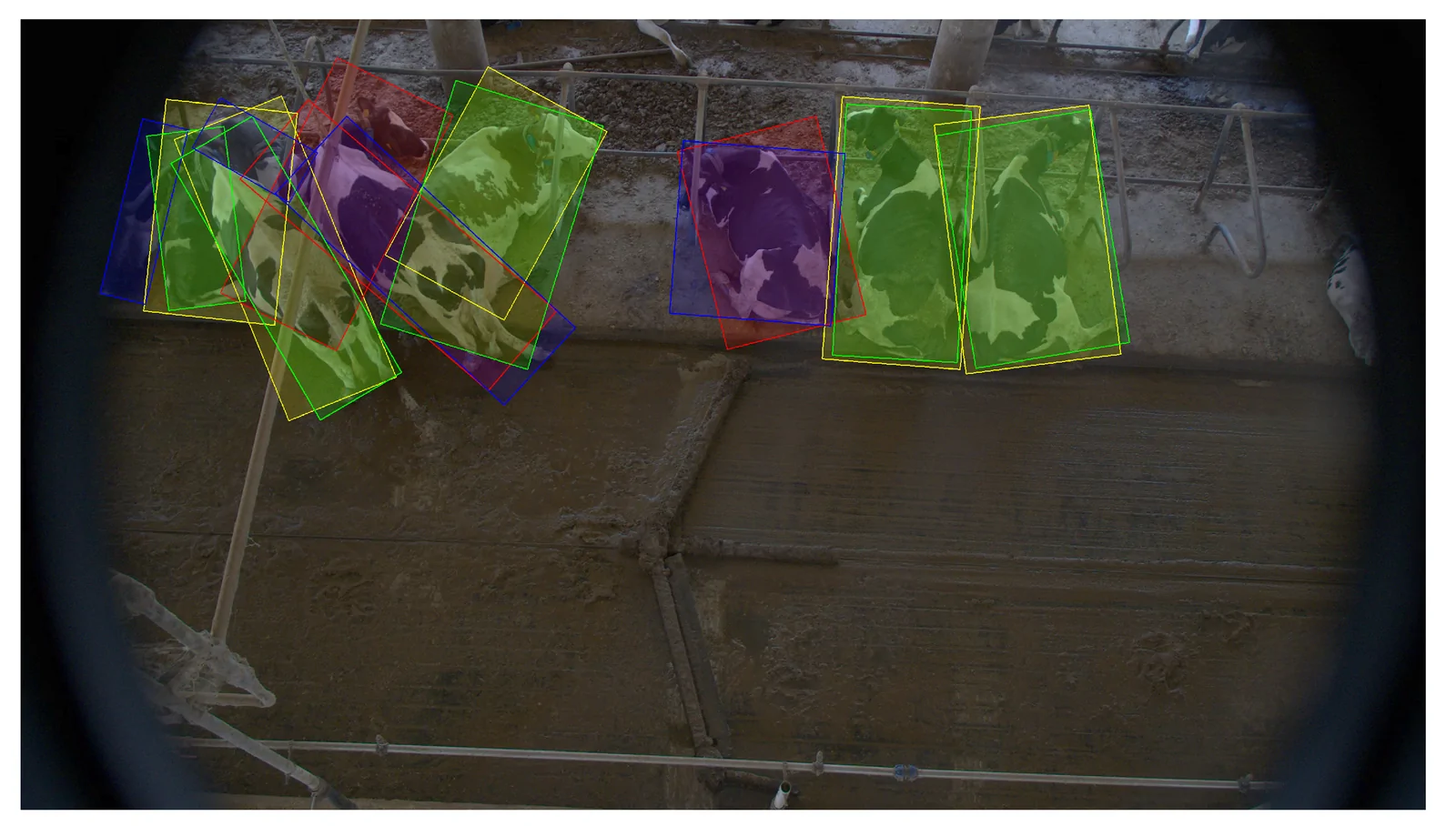
Example of severe occlusion and ambiguous cow poses (top left corner). Such scenarios create annotation ambiguity for both human annotators and automated methods. **Legend:** Yellow = correctly matched EMA ground-truth OBB (True Positive); Green = correctly matched SAM prediction OBB (True Positive); Red = EMA ground-truth OBB missed by SAM (False Negative); Blue = SAM false-positive OBB (False Positive).

Figure 5 shows a challenging scenario with high occlusion where cows’ head orientations do not match their natural body positions. In such cases, determining the exact number of distinct cows becomes ambiguous for both human annotators and SAM 3, potentially leading to disagreements that affect matching statistics.

##### Unmatched Pairs

When corresponding ground truth and SAM 3 detections exist but their overlap falls below both acceptance criteria (IoU < 0.5 and IoM < 0.9), the pair is not matched. Consequently, the ground truth bounding box remains unmatched and is counted as a false negative, while the SAM 3 bounding box is counted as a false positive. This mismatch incurs a two-sided penalty, degrading both precision and recall simultaneously. Since precision consistently exceeded recall in our experiments, false positives were less frequent than false negatives, indicating that SAM 3 detections are generally reliable, while a subset of ground truth instances remained unmatched.

##### Other Limitations

**Figure 6:**
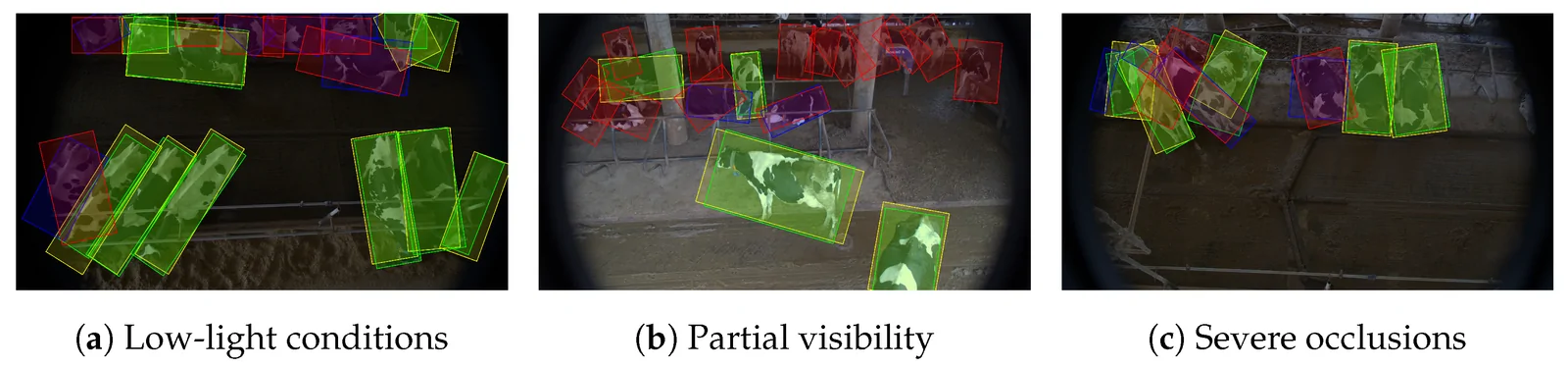
Failure cases of SAM 3 annotations under challenging conditions. Visualization of annotation errors in three difficult scenarios: (**a**) low-light evaluation showing missed detections and misaligned orientations due to reduced visibility and occlusion; (**b**) partial visibility evaluation demonstrating numerous false negatives when cattle are only partially visible; and (**c**) severe occlusion evaluation showing clustered overlapping cattle with heavy infrastructure obstruction. **Legend:** Yellow = correctly matched EMA ground-truth OBB (True Positive); Green = correctly matched SAM prediction OBB (True Positive); Red = EMA ground-truth OBB missed by SAM (False Negative); Blue = SAM false-positive OBB (False Positive).

Our evaluation reveals other challenges that limit the generalization of automated annotations across diverse farm conditions. Figure 6 illustrates three critical failure modes: (1) Low-light conditions where reduced illumination combined with occlusion causes SAM to misalign bounding box orientations and miss cattle instances entirely; (2) Partial visibility where cattle are only partially visible within the frame, leading to substantial false negatives as SAM struggles to detect incomplete animals; and (3) Severe occlusions where multiple cattle overlap or are heavily obscured by infrastructure, creating complex spatial arrangements that confound the model. These scenarios highlight the boundary conditions of the current approach and underscore the need for further development before deployment in truly unconstrained farm environments.

### 5.2. Comparing YOLO Models Fine-Tuned on Ground Truth vs. SAM 3 Annotations

Having established that SAM 3 produces high-quality annotations, we now evaluate whether these automated annotations can serve as reliable training data for task-specific detectors. We trained YOLO11-OBB models on two annotation sources: training subsets of manual ground truth (EMA_1096) and automatic SAM 3 (SAM_1096) annotations—and evaluated both on the same test subset of 165 images with ground truth annotation (EMA_1096) to enable controlled comparison. To account for variability caused by stochastic training, each experimental condition was trained ten times using seeds 0–9. Table 5 reports the mean and standard deviation of all metrics across the ten runs.

The small standard deviations indicate that the results were stable across the ten training runs. The model trained on ground truth annotations shows only slightly higher performance across all metrics, except mAP@0.5–0.95, a comprehensive metric that averages performance across IoU thresholds from 0.5 to 0.95, providing a more rigorous evaluation of localization quality than single-threshold metrics. Therefore, SAM 3-trained model achieves competitive results while requiring zero hours of manual annotation effort.

These results provide evidence that automated SAM 3 annotations can serve as a viable alternative to manual annotations for training cattle detection models, particularly when annotation resources are limited or when scaling to larger datasets.

The last row of Table 5 reports results for a model trained on the larger SAM_6295 dataset and evaluated on the EMA_1096 ground truth test subset of 165 images. Note that these 165 images were excluded from SAM_6295 for the purpose of training, to ensure no data leakage. Training on this larger automated dataset yields slightly improved performance across all metrics compared to SAM_1096, with mAP@0.5–0.95 increasing from 0.8432 to 0.8551. This improvement demonstrates that scaling automated annotation can enhance detection performance, making large-scale dataset creation with SAM 3 a practical strategy for improving detector quality.

### 5.3. Effect of Training Set Size on Detection Performance

To examine how training set size affects detection performance when using automated annotations, and to determine how many automatically annotated images in the training set are required to achieve results comparable to training on the manual annotations, we conducted a systematic study. The EMA_1096 dataset served as the ground truth test set for all experiments. Note that in these experiments, the test size is comparably larger (1096 images) versus the test subset of 165 images used in the previous experiment.

We excluded images contained in EMA_1096 from SAM_6295, leaving 5199 unique images available for training. These were split into training (85%, 4419 images) and validation (15%, 780 images) subsets. We then trained YOLO11-OBB models on progressively larger fractions of the 4419-image training set, ranging from 2% ( 88 images) to 100% (4419 images), with all models evaluated on the whole EMA_1096 ground truth set. This design isolates the effect of training set size while maintaining consistent evaluation.

**Figure 7:**
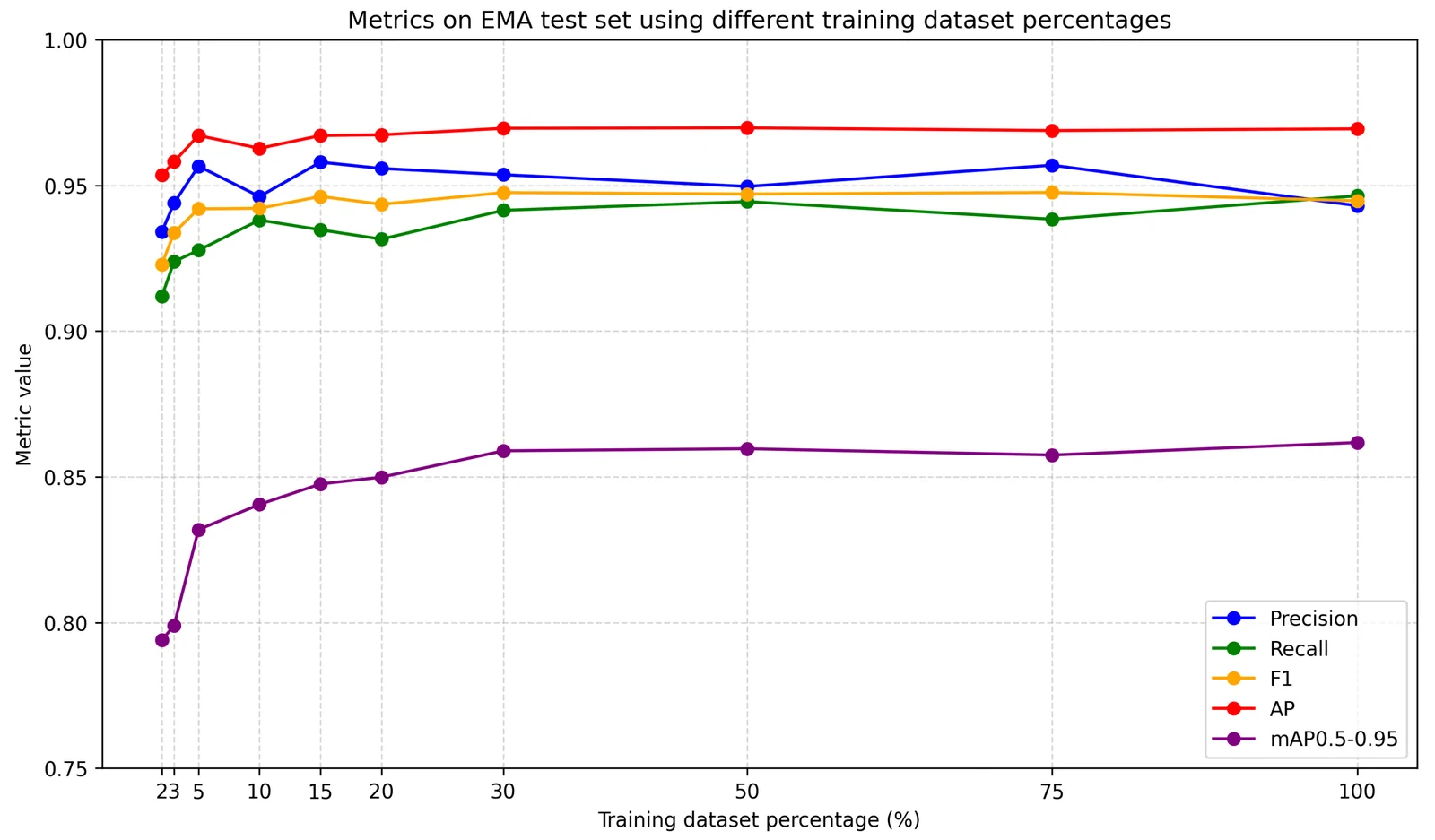
Detection performance metrics as a function of SAM 3 training dataset percentage. All models were evaluated on the same ground truth dataset. The rapid initial improvement and subsequent plateau demonstrate that only a small fraction of automated annotations is needed to achieve high detection accuracy.

Table 6 and Figure 7 present the results.

## 6. Discussion

Several important observations emerge:

- Rapid initial improvement: Performance metrics improve substantially even with very small training set fractions. At just 2% of the training data, the model achieves precision of 0.934, recall of 0.912, and F1 of 0.923.
- Strong performance with minimal data: By 5% of the training set, the model reaches precision of 0.957, recall of 0.928, F1 of 0.942, and mAP@0.5–0.95 of 0.832—approaching the performance of models trained on the manual annotations.
- Diminishing returns: Beyond approximately 30% of the training data, metrics plateau, with only marginal improvements as dataset size increases. The mAP@0.5–0.95 stabilizes around 0.86, indicating that additional training data provides limited benefit once the detector has adapted to the visual characteristics of barn environments and cow appearances.
- Full dataset performance: The model trained on 100% of SAM_6295 annotations achieves precision of 0.943, recall of 0.946, F1 of 0.945, and mAP@0.5–0.95 of 0.862, demonstrating robust detection capability.

These results have several practical implications. The fact that even small amounts of SAM 3 annotations enable high-quality detector training validates the reliability of automated annotation for cattle detection. For new deployments, practitioners can prioritize temporal or camera diversity over sheer volume, knowing that a representative subset is sufficient for adaptation. This behavior contrasts with training detectors from scratch, where performance typically scales more linearly with dataset size. The combination of pretrained weights, automated high-quality annotations from SAM 3, and the constrained visual domain of barn environments enables efficient detector training with minimal manual annotation effort.

### 6.1. Summary of Experimental Findings

Our experimental evaluation demonstrates several key findings within the scope of the present dataset. We note that these findings are based on one dataset and may depend on the sampled images. Therefore, the following conclusions apply to the tested conditions and should not be generalized to new farms or unseen camera conditions without further validation:

1. SAM 3 produces reliable annotations: With appropriate confidence (0.6) and minimal relative area (0.05) thresholds, with matching criterion IoU ≥ 0.5 or IoM ≥ 0.90, SAM 3 achieves precision of 0.911 and recall of 0.948.
2. Automated annotations enable competitive detector training: YOLO11-OBB models trained on SAM 3 annotations achieve 0.9391 precision, 0.9372 recall, and 0.8432 mAP@0.5–0.95, compared to 0.9635, 0.9698, and 0.8506 respectively for models trained on manual annotations.
3. Small training sets are sufficient: Only 5–10% of the SAM 3 training dataset is needed to achieve strong detection performance (F1 > 0.94, mAP@0.5–0.95 > 0.83), with diminishing returns beyond 30%.
4. Dramatic annotation cost savings: Manual annotation requires approximately 400 h of human labor, while SAM 3 processed the same dataset in 1 h with no human intervention required—completely eliminating the manual annotation bottleneck.

These findings collectively demonstrate that foundation models like SAM 3 can be trusted as annotation tools for cattle detection, addressing the annotation bottleneck that has hindered large-scale deployment of vision-based livestock monitoring systems. However, while SAM 3 produces high-quality annotations, deploying it for real-time cattle detection in production environments could be impractical due to computational requirements. SAM 3 requires 19,572 MiB of GPU memory and processes images at 0.58 s per frame, limiting deployment to high-end hardware. In contrast, YOLO11-OBB requires only 344 MiB of GPU memory (57× less) and achieves inference at 0.11 seconds per frame (5.4× faster), making real-time multi-camera monitoring feasible on modest hardware. This motivates our approach: use SAM 3’s superior annotation quality offline to generate training data, then deploy the resulting lightweight YOLO detector for efficient real-time operation. This strategy combines the best of both models—SAM 3’s annotation reliability with YOLO’s computational efficiency. The workflow is now practical: install cameras and collect images → apply SAM 3 for automatic annotation of the representative set of images → fine-tune YOLO on this small subset → deploy tracking, re-identification, and behavior monitoring.

### 6.2. Limitations and Future Work

Key limitations include:

- Limited behavioral annotation: Manual annotations provided within our dataset include only posture labels (standing/lying) and head orientation. Comprehensive behavior monitoring requires additional annotations such as feeding, drinking, walking, and lameness indicators. However, labeling these behaviors on existing automated bounding box annotations is substantially faster than annotating from scratch, as annotators can focus solely on behavioral classification rather than object localization. This two-stage approach—automated detection followed by targeted behavioral labeling—represents a practical compromise between full automation and annotation cost.
- Single-farm dataset: Evaluation is limited to Mitrovac farm during two weeks. Generalization to other farms, breeds, and seasons requires empirical validation.
- Edge cases: Severe occlusion, lighting extremes, and small cow fragments create genuine annotation ambiguity for both manual and automated methods.

Future directions include:

- Validating on multiple farms with different architectures, lighting, and cattle breeds.
- Extending SAM 3 with pose estimation and action recognition for comprehensive behavior annotation.
- Integrating automated detection with tracking and re-identification to validate end-to-end pipeline performance.
- Investigating whether head orientation and posture can be automatically inferred from segmentation masks or pose estimates.

## 7. Conclusions

This work addresses a critical bottleneck in deploying vision-based cattle monitoring systems: the annotation burden required to train robust detectors. We demonstrate that foundation models, specifically SAM 3, can generate reliable training annotations that compete with manual labeling while eliminating manual annotation effort.

Our systematic evaluation on our dataset establishes three key findings:

1. Foundation models produce trustworthy annotations: SAM 3 achieves 0.911 precision and 0.948 recall, validating its use as an automated annotation tool.
2. Automated annotations enable competitive detector training: YOLO11-OBB models trained on SAM 3 annotations achieve 0.941 precision and 0.847 mAP@0.5–0.95, comparable to manually trained models, while significantly reducing annotation time.
3. Minimal training data is sufficient: Only 5–10% of the SAM_6295 dataset is needed to achieve strong detection performance, enabling rapid adaptation to new farm environments.

These findings indicate that automated annotation shows promise for reducing reliance on manual labeling in cattle detection under the tested conditions, potentially lowering deployment costs and facilitating scalable cattle monitoring systems. However, broader validation is required to confirm its applicability across varied operational settings. Detection serves as the foundation for tracking, re-identification, and behavior analysis—essential components of comprehensive precision livestock farming.

The EMA dataset, comprehensive evaluation framework, and trained models are publicly available to support further research and practical deployment of vision-based cattle monitoring systems.

## Figures and Tables

**Table 1:** Comparison of publicly available cattle datasets for overhead/top-down monitoring.

Dataset	Images/Frames	Cows	Box Type	Setting	Identity	Behavior
OpenCows2020 [6]	7043	46	AABB	Barn + UAV	Yes	No
Cows2021 [7]	10,402	186	OBB (torso only)	Barn	Yes	No
MultiCamCows2024 [8]	101,329	90	Tracklets	Barn (multi-cam)	Yes	No
CVB [9]	502 clips	8	AABB	Outdoor field	Yes	11 classes
CBVD-5 [10]	206,100	107	AABB	Barn/ranch	No	5 classes
Koskela et al. [11]	1.7 M	-	Frame-level	Milking station	No	10 classes
COLO [13]	1254	-	AABB	Free-stall barn	No	No
EMA (ours)	6295	-	OBB (full body)	Barn	No	Posture + head

**Table 2:** Organization and experimental purpose of the EMA and SAM 3 annotation subsets.

Subset	Source Images	Images	Annotations	Annotation Type	Visibility	Experimental Purpose
EMA_6295	All images	6295	25,014	Manual	Whole only	Reference for the manual annotation effort required for the complete image set
EMA_1096	Selected common subset	1096	13,119	Manual	Whole + partial	Ground truth for annotation comparison and evaluation of all trained YOLO models
SAM_6295	All images	6295	77,381	SAM 3	Whole + partial	Evaluation of the effect of using a larger automatically annotated training set
SAM_1096	Same images as EMA_1096	1096	13,638	SAM 3	Whole + partial	Direct comparison with EMA_1096 and controlled comparison of models trained using different annotation sources

**Table 3:** Software environment, hardware, and YOLO11-OBB training parameters.

Parameter	Value
Ultralytics version	8.4.15
Python version	3.12.12
PyTorch version	2.7.0 + cu126
CUDA version	12.6
OpenCV version	4.13.0.92
NumPy version	2.4.2
GPU	NVIDIA RTX 4000 Ada Generation (NVIDIA Corporation, Santa Clara, CA, USA)
Initial weights	yolo11n-obb.pt
Input image size (imgsz)	1024
Maximum epochs	100
Batch size	4
Workers	8
Early-stopping patience	100
Optimizer	AdamW, selected automatically
Initial learning rate	0.002
Momentum	0.9
Weight decay	0.0005
Pretrained weights	Enabled
Automatic mixed precision	Enabled
Training seeds	0–9
Dataset split seed	123
Deterministic option	Enabled
HSV hue (hsv_h)	0.015
HSV saturation (hsv_s)	0.7
HSV value (hsv_v)	0.4
Translation (translate)	0.1
Scaling (scale)	0.5
Horizontal flip (fliplr)	0.5
Mosaic (mosaic)	1.0
Final epochs without mosaic	10
Disabled augmentations	Rotation, shear, perspective, vertical flip,
	MixUp, CutMix, and copy-paste

**Table 4:** Precision, recall, F1 score, and average precision across different confidence and MRA threshold combinations obtained by SAM 3, evaluated against ground truth (EMA_1096).

Confidence	MRA	Precision	Recall	F1	AP
0.6	/	0.889	**0.954**	0.920	**0.938**
	0.05	0.911	0.948	**0.929**	0.930
	0.1	0.939	0.918	0.928	0.902
	0.15	0.957	0.877	0.915	0.865
	0.2	0.968	0.836	0.897	0.826
0.75	/	0.951	0.908	0.929	0.892
	0.05	0.956	0.904	**0.929**	0.892
	0.1	0.968	0.883	0.923	0.874
	0.15	0.977	0.849	0.908	0.836
	0.2	0.981	0.816	0.891	0.807
0.9	/	0.992	0.595	0.744	0.591
	0.05	0.992	0.595	0.744	0.591
	0.1	0.992	0.594	0.743	0.591
	0.15	**0.993**	0.590	0.740	0.591
	0.2	**0.993**	0.583	0.735	0.582

**Table 5:** YOLO11-OBB performance when trained on manual (EMA_1096) vs. automated (SAM_1096) annotations, both evaluated on the EMA_1096 ground truth test set. The third row shows results when training on the larger SAM_6295 dataset, demonstrating that scaling automated annotations improves detection performance.

Training Data	Precision	Recall	F1	AP	mAP@0.5–0.95
EMA_1096 (Manual)	**0.9635 ± 0.0035**	**0.9698 ± 0.0047**	**0.9666 ± 0.0025**	**0.9811 ± 0.0015**	0.8506 ± 0.0059
SAM_1096 (Automated)	0.9391 ± 0.0060	0.9372 ± 0.0068	0.9381 ± 0.0026	0.9619 ± 0.0014	0.8432 ± 0.0019
SAM_6295 (Automated)	0.9485 ± 0.0046	0.9393 ± 0.0072	0.9438 ± 0.0021	0.9678 ± 0.0017	**0.8551 ± 0.0036**

**Table 6:** YOLO11-OBB performance on the ground truth dataset when trained on different percentages of the SAM_6295 training dataset. Results demonstrate that even small fractions of automated annotations yield strong detection performance.

Training %	Precision	Recall	F1	AP	mAP@0.5–0.95
2	0.934	0.912	0.923	0.954	0.794
3	0.944	0.924	0.934	0.958	0.799
5	0.957	0.928	0.942	0.967	0.832
10	0.946	0.938	0.942	0.963	0.841
15	0.958	0.935	0.946	0.967	0.848
20	0.956	0.932	0.944	0.967	0.850
30	0.954	0.941	0.948	0.970	0.859
50	0.950	0.944	0.947	0.970	0.860
75	0.957	0.938	0.948	0.969	0.857
100	0.943	0.946	0.945	0.969	0.862

## Data Availability

The original data presented in the study are openly available at https://puh.srce.hr/s/gC2RMdDb2rga2FC (accessed on 21 July 2026).
